# The Importance of Local Context in National Malaria Elimination Efforts

**DOI:** 10.4269/ajtmh.23-0041

**Published:** 2023-02-27

**Authors:** Mark L. Wilson

**Affiliations:** Department of Epidemiology, University of Michigan, Ann Arbor, Michigan

Although global malaria incidence was reduced by more than one-quarter during 2000–2015, progress has mostly stagnated since then.^1^ Concerted interventions have resulted in 21 countries eliminating transmission since 2000,^2^ yet malaria persists in more than 80 countries. These extensive efforts to diminish malaria morbidity and mortality have occurred largely through internationally funded government programs aimed at reducing vector abundance (e.g., environmental management, larviciding), transmission reduction (e.g., insecticide-treated nets [ITNs], indoor residual spraying [IRS], chemoprevention), improving diagnosis (e.g., rapid detection tests), and promptly treating infections (e.g., artemisinin-based combination therapies [ACTs]). In combating malaria, the international donor community spent ∼$500 million annually in the early 2000s, with funding increased to ∼$6 billion each year recently.^3^ Yet despite amplified expenditures and expanded control intervention efforts, global malaria reduction has stalled.

Factors that now threaten effective malaria control and prevention include mounting *Anopheles* vector resistance to insecticides used in ITNs and IRS, increasing *Plasmodium* resistance to ACTs, population growth, decreasing cost-effectiveness of interventions, and infrastructural/implementation challenges.^3^ Evidence suggests the need for more focused interventions that consider local social and environmental conditions, site-specific needs and availability of control options, and people’s knowledge and attitudes about effective antimalaria practices in each setting. In the context of previously successful malaria interventions that have recently faltered, the WHO’s *2022 World Malaria Report* highlighted a distinct priority for surveillance, policy, and need-based interventions that are focused on urban settings.^4^ Although rural socioenvironmental contexts are where most malaria transmission occurs, increasing attention is being paid to malaria in urban settings, particularly because of rural-to-urban movement and rapidly growing populations of people living in cities.

In this issue of the *Journal*, Keys and colleagues^5^ report on people’s knowledge, attitudes, and practices (KAP) concerning malaria after a recent outbreak in urban Santo Domingo, capital of the Dominican Republic (DR). The island of Hispaniola (DR and Haiti) represents the last stronghold of malaria in the Caribbean. The World Bank–defined “urban” population of DR has rapidly increased from 5.27 million (62% of total) in 2000, to 9.25 million (83%) in 2021.^6^ Until recently, malaria in DR was considered mostly “rural,” with ∼1,000 cases reported annually, principally along the border with Haiti. During the past decade, reported malaria cases (all *P. falciparum* or mixed infections) generally declined from 2,482 in 2010 to less than 400 in 2018, but then rebounded to 1,291 in 2019.^4^ During 2015–2020, three-quarters of malaria cases occurred in urban Santo Domingo. The study evaluated malaria KAP among 489 adult residents of 20 randomly selected, high-incidence barrios in Los Tres Brazos (∼1.3 million people in the urban core) and La Ciénaga (pop. ∼483,000 on the periurban fringe). Although these barrios were densely populated, they also had inadequate sewerage and areas of stagnant water. The December 2020 observations occurred at the end of a malaria outbreak in 2019–2020.

Keys et al.^5^ found that more than two-thirds of residents recognized malaria as a local health problem, yet fewer than half knew that transmission is by mosquitoes or could name any effective prevention. Although people’s malaria knowledge was positively associated with local incidence, more than one-third could not name any correct malaria symptom or did not know how malaria was diagnosed. Three-quarters of respondents indicated that they had insufficient mosquito nets for their household, and half of households entirely lacked nets. Indeed, more than half of respondents thought that malaria was not a problem, and three-quarters believed that malaria was not the cause of most fevers. Even though only about half of participants had seen government IRS teams in their neighborhood, the vast majority said they would allow mosquito spraying inside their house. Of those people who had nets, 80% reported that they slept under them, and two-thirds of all respondents had removed stagnant water to reduce mosquitoes near their residence. These results suggest that people in this urban setting were generally underinformed and inadequately supported regarding malaria risk and prevention, yet were open to improving their knowledge and augmenting control and treatment.

The reasons for this sudden urban malaria resurgence are enigmatic but may include increasing human density, rural-to-urban movement, inadequate surveillance, treatment and prevention, and suitable conditions for mosquito vectors.^5^ *Anopheles albimanus* is probably the principle vector,^5^^,^^7^^–^^9^ and it is well established in urban settings of DR and the region.^10^^–^^13^ Although urbanization has been associated with decreased malaria in some contexts,^14^^,^^15^ many diverse environmental conditions and social elements influence transmission risk in each specific urban setting.^16^ *Anopheles albimanus* tends to bite outdoors and early in the evening,^17^^,^^18^ reducing effectiveness of IRS and ITNs.^19^ Mobile populations may acquire infections in rural areas but seek treatment and then transmit to mosquitoes in urban contexts.^20^^,^^21^ In this low-transmission urban setting, limited surveillance and people’s perception that malaria is not an important health concern also may have amplified the outbreak.^5^

Malaria in urban settings is taking on new meaning, as nearly three-quarters of the world’s population will be living in urban settings by 2050, including more than half of those living in malaria-endemic countries.^4^ Analytic tools aimed at identifying and quantifying urban transmission^22^^,^^23^ should be used to enhance surveillance and community-based interventions that are tailored to urban settings. A type of “urban microstratification” is needed to focus control and improve effectiveness.^1^ Interestingly, the WHO and the UN Human Settlements Program have just published a joint “global framework” for addressing malaria in urban areas.^24^ That report highlights how rapid, poorly planned urbanization involving substantial rural-to-urban migration often leads to informal settlements with poor sanitation and unmanaged agriculture, which generate breeding sites for certain *Anopheles* spp. Indeed, new urban environmental and sociobehavioral contexts can select for changes in vector-biting behavior and distributions and heighten chances of new species invading, such as that by the highly urban *An. stephensi* into the African continent.^25^ Frequent rural-to-urban movement may further burden urban health centers with infections that were acquired outside the city. Rates of autochthonous transmission need to be determined, combined with information on local risk factors that are special to urban ecosystems. These steps will allow towns and cities to modify surveillance and prevention strategies to better respond to new and shifting urban malaria risks.^24^
